# Phylogenetic signal in tooth wear dietary niche proxies

**DOI:** 10.1002/ece3.4052

**Published:** 2018-05-03

**Authors:** Danielle Fraser, Ryan J. Haupt, W. Andrew Barr

**Affiliations:** ^1^ Palaeobiology Canadian Museum of Nature Ottawa ON Canada; ^2^ Department of Paleobiology Smithsonian Institution National Museum of Natural History Washington District of Columbia; ^3^ Department of Geology and Geophysics University of Wyoming Laramie Wyoming; ^4^ Center for the Advanced Study of Human Paleobiology Department of Anthropology George Washington University Washington District of Columbia

**Keywords:** dietary niche, Eltonian niche, mesowear, microwear, phylogenetic signal

## Abstract

In the absence of independent observational data, ecologists and paleoecologists use proxies for the Eltonian niches of species (i.e., the resource or dietary axes of the niche). Some dietary proxies exploit the fact that mammalian teeth experience wear during mastication, due to both tooth‐on‐tooth and food‐on‐tooth interactions. The distribution and types of wear detectible at micro‐ and macroscales are highly correlated with the resource preferences of individuals and, in turn, species. Because methods that quantify the distribution of tooth wear (i.e., analytical tooth wear methods) do so by direct observation of facets and marks on the teeth of individual animals, dietary inferences derived from them are thought to be independent of the clade to which individuals belong. However, an assumption of clade or phylogenetic independence when making species‐level dietary inferences may be misleading if phylogenetic niche conservatism is widespread among mammals. Herein, we test for phylogenetic signal in data from numerous analytical tooth wear studies, incorporating macrowear (i.e., mesowear) and microwear (i.e., low‐magnification microwear and dental microwear texture analysis). Using two measures of phylogenetic signal, heritability (*H* ^2^) and Pagel's λ, we find that analytical tooth wear data are not independent of phylogeny and failing to account for such nonindependence leads to overestimation of discriminability among species with different dietary preferences. We suggest that morphological traits inherited from ancestral clades (e.g., tooth shape) influence the ways in which the teeth wear during mastication and constrain the foods individuals of a species can effectively exploit. We do not suggest that tooth wear is simply phylogeny in disguise; the tooth wear of individuals and species likely varies within some range that is set by morphological constraints. We therefore recommend the use of phylogenetic comparative methods in studies of mammalian tooth wear, whenever possible.

## 1 INTRODUCTION

The niche of a species is formally defined as the *n*‐dimensional hypervolume that encompasses the set of biotic and abiotic conditions in which they live (Hutchinson, 1957). Niches can further be refined as scenopoetic or bionomic, which describe the bioclimatic and resource axes of a species niche, respectively (Hutchinson, 1978), and are roughly equivalent to the Grinellian (Grinnell, 1917) and Eltonian (Elton, 1927) concepts of the niche. The Eltonian niche describes the role of a species in a community, with an emphasis on their trophic or dietary niche (Elton, 1927; Soberón, 2007). The Eltonian niche has been of particular interest to ecologists and paleoecologists studying topics including, but not limited to, defining niche space occupation and trophic interactions in the present and past (DeSantis & Haupt, 2014; DeSantis et al., 2015; Rivals & Semprebon, 2006; Semprebon & Rivals, 2007, 2010), determinants of extinction risk (DeSantis, Feranec, & MacFadden, 2009; DeSantis & Haupt, 2014; Fraser, Gorelick, & Rybczynski, 2015; Smits, 2015), and ecosystem changes through time (Fraser & Theodor, 2013; Janis, Damuth, & Theodor, 2000, 2004; Merceron & Madelaine, 2006; Merceron et al., 2004; Ungar, Merceron, & Scott, 2007; Ungar, Scott, Scott, & Teaford, 2007). Because the dietary niches of species cannot always be directly observed, neoecologists and paleoecologists use a variety of proxies. A widely employed set of approaches exploit the fact that teeth wear during feeding. Among mammals, the chewing or occlusal surfaces of the teeth are altered during mastication by both attrition (i.e., tooth‐on‐tooth) and abrasion (i.e., food‐on‐tooth) (as well as chewing and ingestion of other exogenous environmental materials; Fortelius & Solounias, 2000; Hoffman, Fraser, & Clementz, 2015; Janis, 1990). Such tooth wear is informative because the degrees and types of wear present on the occlusal surfaces correlate strongly with the dietary niches of species (Fraser & Theodor, 2011; Janis, 1990), thus allowing inferences about Eltonian niches in the absence of independent observational data.

Tooth wear methods provide information on everything from a lifetime of tooth wear to the last few meals (Davis & Pineda‐Munoz, 2016; Fortelius & Solounias, 2000; Grine & Kay, 1988). For mammals, the two most commonly employed sets of proxies are tooth macrowear (also referred to as mesowear) and microwear (microscopic tooth wear). Analytical tooth wear methods such as mesowear and microwear involve quantification of macroscopic and microscopic features on the occlusal surfaces of teeth in an attempt to characterize the diets of individuals and, in turn, the Eltonian niches of entire species (DeSantis & Haupt, 2014; DeSantis et al., 2015; Donohue, DeSantis, Schubert, & Ungar, 2013; Fortelius & Solounias, 2000; Fraser, Mallon, Furr, & Theodor, 2009; Fraser & Theodor, 2010, 2011, 2013; Fraser, Zybutz, Lightner, & Theodor, 2014; Grine & Kay, 1988; Haupt, DeSantis, Green, & Ungar, 2013; Hedberg & DeSantis, 2017; Semprebon, Godfrey, Solounias, Sutherland, & Jungers, 2004; Solounias, Moelleken, & Plavcan, 1995). Tooth wear data are then typically compared to observed dietary data (e.g., gut contents, fecal contents, personal observations) in a reference population or sample of species (i.e., the training dataset) to create a set of regression coefficients or discriminant functions that can be used to infer the diets of species and individuals for which independent dietary data are unavailable (Barr & Scott, 2014; Fraser & Theodor, 2011). Many studies have found analytical tooth wear methods to be statistically powerful means of dietary inference (Donohue et al., 2013; Fortelius & Solounias, 2000; Fraser & Theodor, 2011; Haupt et al., 2013; Hedberg & DeSantis, 2017; Semprebon et al., 2004; Solounias & Semprebon, 2002); rates of correct classification of species with known diets are often fairly high (~80% or higher, depending on the study) (Fraser & Theodor, 2011), allowing dietary inference from tooth wear with a reasonable degree of confidence.

Tooth wear studies do not assume that all individuals in a particular taxon have the same realized diet, but rather aim to infer individual diet directly from the mechanical traces left on occlusal surfaces. Thus, differences in diet among individuals, even among different life stages of the same individual, can be quantified using analytical tooth wear methods (Calandra & Merceron, 2016; DeSantis, Field, Wroe, & Dodson, 2017; DeSantis & Haupt, 2014; Rivals, Mihlbachler, & Solounias, 2007; Rivals, Schulz, & Kaiser, 2008; Rivals & Semprebon, 2006). Numerous studies restrict their dietary analysis to one or a few closely related taxa, thus making the implicit assumption that comparisons cannot be made across highly disparate species (e.g., DeSantis & Haupt, 2014; Haupt et al., 2013). However, tooth wear is also used for the inference of average species’ diets. In these cases, a number of individuals are sampled in hopes of averaging‐out individual dietary differences (Fortelius & Solounias, 2000; Solounias & Semprebon, 2002). Both mesowear and microwear are then frequently compared across broad taxonomic groups, typically within a dietary guild (e.g., herbivores; Christensen, 2014; Fortelius & Solounias, 2000; Solounias & Semprebon, 2002). Mesowear and microwear are, however, often referred to as “taxon free”; that is, there is an implicit and sometimes explicit assumption that the taxon to which an individual belongs has no bearing on their tooth wear. As a result, phylogenetic relatedness is rarely accounted for in studies of extant and extinct mammal diets (but see Mihlbachler & Solounias, 2006 and Fraser et al., 2014), which may be confounding if phylogenetic niche conservatism (PNC) is widespread (Olalla‐Tárraga, González‐Suárez, Bernardo‐Madrid, Revilla, & Villalobos, 2017).

Phylogenetic niche conservatism implies that evolutionary change along niche axes is slow (Cooper, Jetz, & Freckleton, 2010; Olalla‐Tárraga et al., 2017). If PNC is the rule, over evolutionary time, the Eltonian niches of closely related species should remain similar. Evolutionary conservatism has been observed for traits that are highly correlated with species’ trophic niches, including body size (Fraser & Lyons, 2017; Perez‐Barberia & Gordon, 1999, 2001; Pineda‐Munoz, Evans, & Alroy, 2016; Smith et al., 2004) and some estimates of trophic level (Olalla‐Tárraga et al., 2017). Furthermore, rates of evolutionary transition among trophic levels (i.e., omnivore, herbivore, and carnivore) are asymmetric and low, implying reduced dietary lability over evolutionary time, especially among herbivores and carnivores (Price, Hopkins, Smith, & Roth, 2012). Conservatism of morphological traits associated with feeding and thus trophic level as well as slow rates of evolutionary transition among trophic levels suggests that closely related species should have similar (although not necessarily identical) patterns of tooth wear (in fact, we make this implicit assumption when we analyze subsets of individuals to infer diet for species as a whole). Therefore, we hypothesize that dietary inferences from tooth wear are biased by phylogenetic relatedness and that tooth wear dietary proxies show strong phylogenetic signal. If trophic PNC is common among mammals and tooth wear is shown to be nonindependent of phylogenetic relatedness, the assumption of taxon independence when inferring species’ diets using tooth wear may be incorrect.

An assumption of phylogenetic independence may become particularly problematic when investigators use statistical or exploratory methods that assume data point independence to distinguish among species in different dietary guilds or trophic levels. The statistical consequences of nonindependence are discussed at length elsewhere (Felsenstein, 1985; Freckleton, Harvey, & Pagel, 2002; Garland, Bennett, & Rezende, 2005; Garland, Harvey, & Ives, 1992; Price, 1997). However, we reiterate that statistical nonindependence of data points, such as occurs when a trait of interest is highly phylogenetically conserved, can lead to bias in the estimation of *p*‐values, increases in type I error rates (i.e., false positives) (Barr & Scott, 2014; Rohlf & Hansen, 2006), and thus unjustified confidence in the utility of a dietary metric for distinguishing species with different Eltonian niches. Furthermore, nonindependence of data points can lead to overestimates of discriminability among groups when using data exploration techniques such as discriminant function analysis.

Herein, we test for phylogenetic signal in tooth wear metrics, specifically tooth macro‐ and microscopic wear. We include tooth mesowear (Fortelius & Solounias, 2000), low‐magnification tooth microwear (Solounias & Semprebon, 2002), and dental microwear textures (Ungar, Brown, Bergstrom, & Walker, 2003) from a variety of published sources (Appendix S1). A finding of high phylogenetic signal in the tooth wear data would suggest that statistical compensation is required to fully understand the degree to which diet can be inferred.

### 1.1 Analytical tooth wear methods

The most widely employed methods for dietary inference include mesowear, the visual scoring of macroscopic tooth wear (Fortelius & Solounias, 2000; Fraser et al., 2014; Kaiser & Fortelius, 2003; Kaiser & Solounias, 2003), and microwear, the quantification of microscopic marks on the chewing surfaces of the teeth at either low or high magnification (Grine & Kay, 1988; Merceron, Blondel, Bonis, Koufos, & Viriot, 2005; Semprebon et al., 2004; Solounias & Semprebon, 2002; Ungar et al., 2003). Both mesowear and microwear have proven to be extremely useful tools for inferring the dietary preferences of extant and extinct mammals (and nonmammals) from numerous families (DeSantis & Haupt, 2014; DeSantis et al., 2015; Donohue et al., 2013; Fortelius & Solounias, 2000; Fraser & Theodor, 2010, 2011, 2013; Fraser et al., 2009, 2014; Grine & Kay, 1988; Haupt et al., 2013; Hedberg & DeSantis, 2017; Semprebon et al., 2004; Solounias et al., 1995).

The mesowear method, as proposed by Fortelius and Solounias (2000), approximates the diets of herbivorous “hoofed mammals” (including artiodactyls, perissodactyls, with newer iterations also including proboscideans) by quantifying the relative amounts of attritional and abrasive wear apparent on the cusps of the cheek teeth (premolars and molars). Mammalian mastication is driven by asymmetric contraction of the masseter, pterygoideus, and temporalis muscles on the working and balancing sides, which produces a transverse movement of the mandible (Janis, 1979; Rensberger, 1973; Smith, 1993). During the chewing stroke, the cheek teeth experience both attrition and abrasion (Fortelius & Solounias, 2000). Attrition occurs during the interaction of opposing wear facets and results in cusp sharpening as the facets slide past one another. Abrasion occurs during the interaction of the teeth with ingested items including food particles, resulting in the dulling of the cusps (Fortelius & Solounias, 2000).

The original Fortelius and Solounias (2000) mesowear method involves visual categorization of tooth cusp relief (the height difference between the intercusp valleys and the tip of the cusp) and shape (sharp, round, blunt) (Fortelius & Solounias, 2000). Individuals that experience high attrition (browsers) tend to have high relief and sharp cusps while individuals that experience high abrasion (grazers) have low relief and blunt cusps. Intermediate or mixed feeders (10%–90% grass consumption) tend to have low‐ or high‐relief cusps with rounded tips, depending on whether their diet is dominated by grasses or browse (Fortelius & Solounias, 2000). Mesowear yields fairly high rates of correct classification (~60%–80%) of ruminant artiodactyls among the dietary categories of browser, grazer, and mixed feeders (Fraser & Theodor, 2011; Fraser et al., 2014).

Microwear is the quantification of microscopic wear features on the teeth that result from masticating different foods with different physical properties (Solounias & Semprebon, 2002). Traditionally, microwear analysis involves the quantification of the numbers of pits and scratches on the tooth surface within predefined counting areas or grids as identified by one or more human observers (Grine & Kay, 1988; Solounias & Semprebon, 2002). Pits are thought to be formed as a result of enamel on enamel contact during which small fragments of enamel are chipped away, while scratches are thought to be formed by abrasive items such as phytoliths or exogenous grit being scraped across the enamel surface (Hoffman et al., 2015; Solounias & Semprebon, 2002). Traditional microwear analysis may be carried out at either high (Grine & Kay, 1988) or low magnification (Semprebon et al., 2004; Solounias & Semprebon, 2002). The low‐magnification method has been widely employed due to low cost and the accessibility of light microscopes, whereas the high‐magnification method requires a scanning electron microscope (SEM). Low‐magnification microwear methods yield intermediate results of successful dietary classification for ruminants (~64%) but, in combination with other dietary metrics such as mesowear, yield very high rates of correct classification (~90%) (Fraser & Theodor, 2011).

Studies of tooth microwear have also grown to include three‐dimensional textural analysis of tooth surfaces at high magnification (Ungar, Simon, & Cooper, 1991). A popular implementation of this approach is known as dental microwear textural analysis (DMTA), for which the tooth surface is scanned across the *z*‐axis using a white‐light confocal profiler generating a point cloud matrix (Scott et al., 2006). Point cloud matrices are then analyzed using scale‐sensitive fractal analysis, which operates on the idea that apparent length, area, and volume can change at varying observational scales, and that these changes correlate to the material properties of consumed food (Ungar et al., 2003). For example, a surface that seems smooth at the coarse scale of a human observer, such as a rug, might seem rough and varied at the scale of an ant. Implementation of DMTA has been limited by the low number of available microscopes and the high cost of the analytical software required to compute the necessary textural values (SurFract, Norwich, VT, USA). However, DMTA is shown to be an effective method for differentiating a wide array of mammalian taxa based on dietary preference including artiodactyls (Scott, 2012; Ungar, Merceron, et al., 2007; Ungar, Scott, et al., 2007; Ungar et al., 2012), shrews (Withnell & Ungar, 2014), primates (Scott, Teaford, & Ungar, 2012), carnivorans (Desantis, Schubert, Scott, & Ungar, 2012; Schubert, Ungar, & DeSantis, 2010; Ungar, Scott, Schubert, & Stynder, 2010), marsupials (Prideaux et al., 2009), and xenarthrans (Haupt et al., 2013).

## 2 MATERIALS AND METHODS

Mesowear and microwear data were collected from the literature (Appendix S1). Mesowear data are heavily biased toward “hoofed mammals,” given that they are devised for dietary inference in selenodont and lophodont perissodactyls and artiodactyls. Although newer mesowear methods have been developed for proboscideans (Saarinen et al., 2015), the phylogenetic breadth of these datasets is too small for the analyses used herein. Furthermore, given that Proboscidean mesowear is scored on a different scale, the data cannot easily be integrated into other existing mesowear datasets. Similarly, we have not included the mesowear III method (Solounias, Tariq, Hou, Danowitz, & Harrison, 2014) because the training dataset is too small for a statistically robust analysis of phylogenetic signal. Unlike mesowear, the microwear data (low magnification and textural) encompass mammals from most dietary guilds and most major clades (Appendix S1).

The Fortelius and Solounias (2000) mesowear method involves visually categorizing the cheek teeth based on two variables: cusp relief (the height difference between the intercusp valleys and the tip of the cusp) and cusp shape (Fortelius & Solounias, 2000). Cusp relief categories are high and low [cutoffs are discussed in Fortelius and Solounias (2000)]. The cusp‐shape categories are sharp, rounded, and blunt. Sharp cusps are defined as those with little or no rounding between the mesial and distal wear facets. Rounded cusps are defined as those with a smooth cusp tip intervening between the mesial and distal wear facets. Blunted cusps are defined as those in which the wear facets had been worn away (Fortelius & Solounias, 2000). Modifications of the Fortelius and Solounias (2000) mesowear method involve assigning each individual tooth a number based upon cusp shape and cusp relief according to two methods. Teeth that show high relief and sharp cusps are then assigned a value of 0, those with high relief and round cusps are assigned a value of 1, those with low relief and rounded cusps are assigned a value of 2, and those with low or negative relief and blunted cusps are assigned a value of 3. Mesowear scores of individuals are then averaged to produce a species average (Rivals & Semprebon, 2006; Rivals et al., 2007).

For the second mesowear scoring method of Kaiser, Brasch, Castell, Schulz, and Clauss (2009), the cusps are categorized in the same way as Fortelius and Solounias (2000) except that they add an additional mesowear category, low relief and sharp cusps. Teeth that show high relief and sharp cusps are assigned a value of 0, those with high relief and round cusps are assigned a value of 1, those with low relief and sharp cusps are assigned a value of 2, those with low relief and rounded cusps are assigned a value of 3, and those with low or negative relief and blunted cusps are assigned a value of 4 (Kaiser et al., 2009).

The “low‐magnification” microwear (LMM) method was developed by Solounias and Semprebon (2002) and uses light stereomicroscopy (rather than SEM) to examine microwear at comparatively low magnification, thus allowing for large numbers of samples to be processed relatively quickly. The original LMM method involves visualizing tooth casts under a standard light stereomicroscope at 35× magnification utilizing oblique lighting and evaluating the number of pits and scratches on the enamel surface in a 0.4 × 0.4 mm area. Some newer implementations have increased the magnification to 70× (Townsend & Croft, 2008), but we have not included these data in our analyses for reasons of comparability. Photographic methods have been introduced (Fraser et al., 2009; Merceron et al., 2005) but have not been applied widely enough for inclusion.

The DMTA method was developed by Ungar et al. (1991) and uses a white‐light confocal profile to generate and quantify a 3D point cloud of the surface being scanned (Scott et al., 2006). For each analysis, the profiler scans at 100× magnification four adjacent fields of 104 × 138 μm^2^ each, spaced laterally by 0.18 μm and with a vertical resolution >0.05 μm in order to be comparable to SEM‐based microwear (Ungar, 2015). However, attempts to compare DMTA photosimulations to other types of microwear have produced mixed results (DeSantis et al., 2013). Using scale‐sensitive fractal analysis (SSFA), quantified length, area, and volume scale measurements are calculated, which are the values used for comparison and analysis across and between taxa (Articus, Brown, & Wilhelm, 2001; Brown & Siegmann, 2001; Pedreschi, Aguilera, & Brown, 2000). While SSFA produces over a dozen textural variables (Scott et al., 2006), only three are consistently reported in the literature. The length‐scale variable, anisotropy (*epLsar*), represents how similarly directionally aligned features of similar length are to one another, with higher anisotropy indicating more aligned features (Ungar et al., 1991). Consumption of tough foods (e.g., grass, muscle) may yield more anisotropic surfaces (Scott et al., 2012). The area‐scale variable, area‐scale fractal complexity (*Asfc*), is a measure of how surface area changes as a function of scale, where the resultant *Asfc* value is the curve of the steepest part of the slope (Scott et al., 2006). Higher *Asfc* values indicate a more “pitted” surface thought to be caused by the consumption of brittle foods (e.g., nuts, bones) (Scott et al., 2005; Ungar, Scott, et al., 2007; Ungar, Merceron, et al., 2007; Ungar et al., 2003). The volume scale variable, textural fill volume (*Tfv*), is measured by filling the scanned area with course (10 μm) and fine (2 μm) cuboids. The course volume is then subtracted from the fine volume, yielding only the volume of the textural features and canceling out any overall shape or curve of the broader scanned area (Scott et al., 2006). Higher *Tfv* is associated with chewing of harder food items (e.g., chitin and calcite shells, grit) (Scott et al., 2012). Additional textural features not often reported in the literature are described in Scott et al. (2006).

To summarize the multivariate DMTA data, we performed a principal component analysis (PCA) on *epLsar*,* Asfc*, and *Tfv* and extracted the species scores on the first and second principal components for further analysis.

We also extracted mammal dietary information from the EltonTraits 1.0 database (Wilman et al., 2014). The EltonTraits diet data were combined into six categories: the percentage of the diet composed of (1) invertebrates, (2) vertebrates, (3) fruits, (4) nectar, (5) seeds, and (6) other plant materials (e.g., leaves and stems). Because the data must sum to 100% and are thus inappropriate for PCA, we transformed the data into *z*‐scores (Pineda‐Munoz, Lazagabaster, Alroy, & Evans, 2017). We calculated the *z*‐scores as the difference between individual values and the group mean divided by the standard deviation. We extracted the species scores on the first and second principal components for further analysis.

### 2.1 Testing for phylogenetic signal

We used the phylogeny of Faurby and Svenning (2015) for all of the analyses of phylogenetic signal. The number of species included in each analysis of phylogenetic signal is shown in Table 1.

**Table 1:** Summary of phylogenetic signal tests as measured using heritability (*H* ^2^) and Pagel's λ

Dietary metric	No. of species	*H* ^2^ lower 95% confidence limit	*H* ^2^ upper 95% confidence limit	*H* ^2^ mean	λ
Diet (PC1)	5,040	0.992	0.994	0.993	0.993
Diet (PC2)	5,040	0.995	0.996	0.996	0.998
Mesowear (Fortelius)	75	0.876	0.995	0.966	0.952
Mesowear (Kaiser)	73	0.530	0.999	0.989	0.968
Low‐magnification microwear	126	0.247	0.847	0.617	0.537
DMTA (PC1)	77	0.850	0.984	0.954	0.938
DMTA (PC2)	77	0.763	0.997	0.957	0.934

We used two measures of phylogenetic signal: (1) phylogenetic heritability or *H* ^2^ sensu (Lynch, 1991) and (2) Pagel's λ (Freckleton et al., 2002; Pagel, 1992, 1999). We do not use *p*‐values because the traditional cutoffs between significant and nonsignificant (.05 or .001) are arbitrary and thus uninformative for our analyses.

*H* ^2^ is a measure of the phylogenetic heritability of a trait because it is an estimate of the degree to which phylogenetically related species can be used to predict the phenotype of each other (Lynch, 1991). *H* ^2^ is calculated as:

$$H^{2}=\frac{{\hat{\sigma }}_{\alpha }^{2}}{{\hat{\sigma }}_{T}^{2}}$$

where ${\hat{\sigma }}_{\alpha }^{2}$ is the additive component of the trait variance (the phylogenetic effect or component that is transmitted to descendants) and ${\hat{\sigma }}_{T}^{2}$ is the estimated total variance of the trait mean (the sum of the additive variance and residual variance). We calculated *H* ^2^ using a phylogenetic generalized linear mixed‐effects modeling in the MCMCglmm R package (Hadfield, 2010). We included phylogeny as a random effect and used an inverse Wishart prior for the variances and a normal prior for the fixed effects. The parameters for the inverse Wishart are *V* and ν, the variance and degree of belief for *V*, respectively. For all models, we used an uninformative prior with the following parameters: *V *= 10^−6^, ν = −1. MCMCglmm uses a normal distribution for the fixed effects, which we considered appropriate. We ran the MCMC for 2,600,000 iterations with a burn‐in of 600,000 iterations. We subsampled the MCMC at an interval of 2,000 iterations. In *R*,* H* ^2^ was then calculated by dividing the posterior distribution of covariance matrices for the random effect (phylogenetic effect) by the sum of the covariance matrices for the random and fixed (tooth wear) effects. We report the mean and upper and lower 95% confidence limits.

We also estimated Pagel's (1999) λ using fitContinous in the Geiger R package (Harmon, Weir, Brock, Glor, & Challenger, 2008). Pagel's λ is a branch‐length transformation parameter that is iteratively estimated using maximum likelihood (ML) from a set of tip data and a phylogenetic topology. When λ = 0, all tips of the phylogeny are equal to the root‐tip distance, and thus, the topology is equivalent to a star phylogeny. As all tip branch lengths are the same, a ML value of 0 for λ indicates a situation in which there is no relationship between tip data and branch lengths in the tree. When λ = 1, the internal branches of the phylogenetic tree are left alone. Therefore, a ML value of λ = 1 applies to tip data that are directly inversely related to the branch lengths connecting tip taxa. This is equivalent to a Brownian motion model of character evolution along the phylogenetic topology.

### 2.2 Comparing phylogenetic and nonphylogenetic tests of discriminability

To demonstrate the impact of using phylogenetic comparative methods on discriminability among species with differing dietary niches, we used phylogenetic discriminant function analysis (PDFA) and quadratic discriminant function analysis (QDFA). We chose to analyze the LMM dataset for this purpose because microwear is comparable among a large set of taxa (unlike mesowear, which can be quantified only for selenodont artiodactyls and lophodont perissodactyls). Dietary categorization was based on the Elton Traits 1.0 database (Wilman et al., 2014). Carnivores were defined as species relying primarily on vertebrates and invertebrates (including insectivores). We combined vertebrate and invertebrate carnivores primarily because very few species specialize exclusively on one or the other, excepting mammalian hypercarnivores; and we wanted to avoid arbitrary cutoffs as much as possible. Herbivores were defined as species relying primarily on plants (including fruits). Finally, omnivores were defined as species relying on any mix of vertebrates, invertebrates, and plants. We recognize that mammal dietary data can be classified in a variety of ways but employment of a different classification system would not alter our prediction that nonphylogenetic methods overestimate discriminability among dietary guilds.

Quadratic discriminant function analysis is used for discrimination of groups, in this case dietary guilds, based on a multivariate dataset (the training dataset). QDFA identifies the combination of variables (quadratic functions) that achieves maximum separation among the defined groups in the training dataset (Hammer & Harper, 2006). QDFA differs from linear DFA in allowing for unequal variance–covariance matrices among classes (i.e., dietary guilds). Classification is made by assigning observations to the class with the closest centroid using the Mahalanobis distance. The utility of the resulting set of discriminant functions is assessed by the rate at which observations are correctly grouped into their original class. A high rate of correct classification means that species with unknown diets can be classified with a reasonable degree of confidence.

Phylogenetic discriminant function analysis relies on an approach called flexible discriminant analysis, which takes advantage of the fact that linear discriminant analysis (LDA) is equivalent to canonical correlation analysis and thus reduces to a multiresponse linear regression (Hastie, Tibshirani, & Buja, 1994). When implementing FDA, portions of the LDA calculation are replaced by least squares regression (Hastie et al., 1994), thus also allowing for the removal of phylogenetic bias (Motani & Schmitz, 2011). Phylogenetic comparative methods such as phylogenetic generalized least squares regression remove phylogenetic bias through a modification of variance–covariance matrices (Grafen, 1989), as is done during PDFA. We implemented PDFA as described in detail by Motani & Schmitz, 2011.

## 3 RESULTS

**Figure 1:**
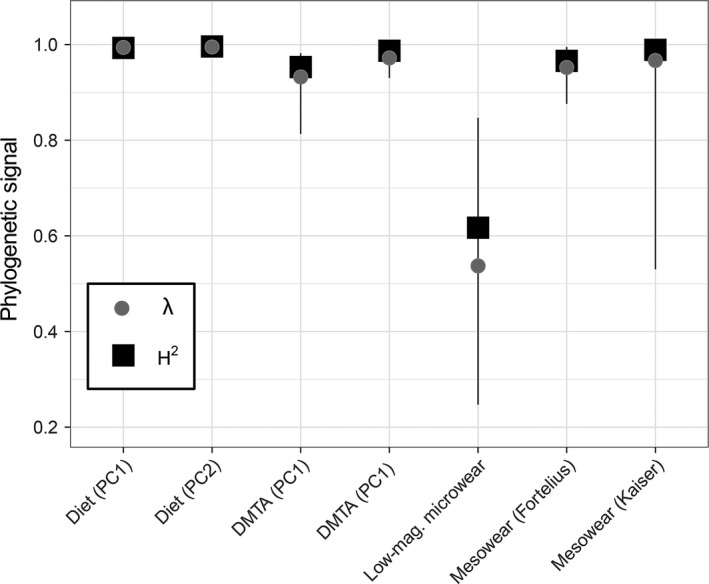
Analytical tooth wear methods show high phylogenetic signal as measured using heritability (*H* ^2^) and Pagel's λ. DMTA stands for dental microwear texture analysis. PC1 and PC2 refer to the first and second principal components, respectively

**Figure 2:**
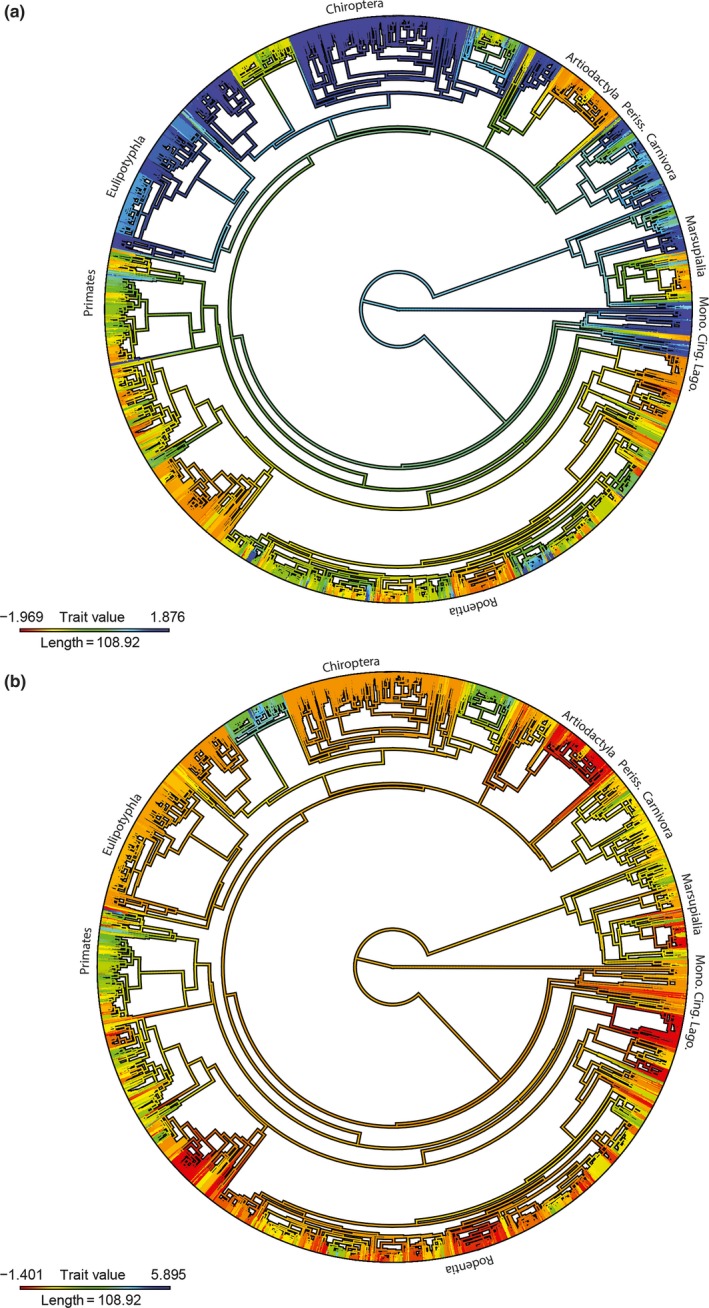
Dietary preference shows phylogenetic clustering that corresponds to the major clades of mammals. Phylogenetic trait maps of the first (a) and second (b) principal components of mammal diet. Darker colors indicate positive values on the principal component axis while warm colors indicate negative values

The first (PC1) and second (PC2) principal components of mammal diet explain a cumulative 50.8% of the variance in the data. PC1 most strongly delineates insectivores and carnivores from species that feed primarily on plant materials. PC2 reflects separation of the frugivores and nectar specialists from other species (Figure S1). Diet PC1 and PC2 show very high values of λ and phylogenetic heritability or *H* ^2^, which suggest a high level of dietary PNC among the taxa examined (Table 1; Figures 1, 2a–b).

Likewise, mesowear (both scoring methods) shows very high values for *H* ^2^ and λ (Table 1; Figures S2–S3). LMM stands out as having the lowest values for both heritability and phylogenetic signal as estimated by λ (Table 1; Figure S4).

PC1 and PC2 of dental microwear textures (DMTA) explain a cumulative 79.2% of the data variance. PC1 reflects separation of species with high anisotropy (*epLsar*) and high‐scale fractal complexity (*Asfc*) as well as textural fill volume (*Tfv*). PC2 largely reflects the separation of species with high textural fill volume from the others (Figure S5). Similar to diet and mesowear, DMTA PC1 and PC2 show very high values of *H* ^2^ and λ (Table 1; Figures S6–S7).

Among the categories of carnivore, herbivore, and omnivore, quadratic discriminant analysis misclassified ~34.5% of species, thus correctly classifying ~65.5% of mammal species for which we compiled microwear data. In contrast, PDFA incorrectly classified ~73.6% of species, thus correctly classifying only ~26.4% of species in the dataset. The high rate of misclassification using PDFA results largely from misclassification of carnivorous and herbivorous species as omnivorous.

## 4 DISCUSSION

Tooth wear is known to reflect everything from the lifetime diet to the last few meals of individual animals and is therefore thought to vary on relatively short timescales (Davis & Pineda‐Munoz, 2016; Teaford & Oyen, 1989); tooth wear shows change throughout the lifetime of individuals as well as variation among individuals, and different populations within species (DeSantis et al., 2009; Fortelius et al., 2002; Rivals & Semprebon, 2006; Scott et al., 2005). For these reasons, tooth wear is often considered to be taxon or phylogeny free, as it is thought to reflect feeding behavior under the implicit assumption that this behavior is highly labile (Blomberg, Garland, & Ives, 2003). Despite the assumption of lability, one of the most common applications of analytical tooth wear methods is for the inference of average species diets (Fortelius & Solounias, 2000; Fraser & Theodor, 2011; Semprebon et al., 2004; Solounias & Semprebon, 2002), an approach that has been applied with considerable success (Donohue et al., 2013; Fortelius & Solounias, 2000; Fraser & Theodor, 2011; Haupt et al., 2013; Hedberg & DeSantis, 2017; Semprebon et al., 2004; Solounias & Semprebon, 2002). We show herein that mammalian diets, summarized at the species‐level, show high phylogenetic signal (Table 1; Figure 2a–b) and that mammals may show some degree of trophic PNC (although it is outside the scope of this paper to definitively test for PNC in mammal diets; Münkemüller, Boucher, Thuiller, & Lavergne, 2015).

If tooth wear proxies are reliable reflections of average species’ diets, as many studies suggest they are (Donohue et al., 2013; Fortelius & Solounias, 2000; Fraser & Theodor, 2011; Haupt et al., 2013; Hedberg & DeSantis, 2017; Semprebon et al., 2004; Solounias & Semprebon, 2002), then logic would dictate that they must show a similar degree of phylogenetic signal. We demonstrate that all of the analytical tooth wear data analyzed herein show strong phylogenetic signal (Table 1; Figure 1); mean *H* ^2^ and Pagel's λ are consistently high for mesowear and microwear. Microwear is thought to be acquired during an animal's last few meals, suggesting a greater degree of lability, but we find similar degrees of phylogenetic signal for mesowear and dental microwear textures (DMTA; Table 1; Figure 1). LMM shows the lowest phylogenetic signal, although we still consider it to be high (Table 1; Figure 1). The difference in phylogenetic signal between LMM and DMTA might reflect (1) the coarser scale at which LMM is quantified, (2) observer bias (i.e., high variability among the studies we included), or (3) that LMM is less reflective of enamel microstructure, which is likely to be phylogenetically inherited. Regardless, as with diet (Table 1; Price et al., 2012), the ways in which mammal teeth wear appear to be conserved among closely related taxa and slow to change on evolutionary timescales. The result is the overestimation of discriminability among mammal species with diverse diets. In the case of LMM, we find a ~40% difference in correct dietary classification between quadratic and PDFA.

We suggest that our finding of high dietary and tooth wear phylogenetic signal among mammals reflects evolutionary conservation of functional traits related to feeding; that is, the traits that determine the foods that individuals can effectively exploit are phylogenetically conserved, these traits constrain the average diets of species, and, in turn, the ways in which the teeth wear during feeding.

Mammal diets are constrained by their feeding morphology (i.e., the form and function of the feeding apparatus) (Mendoza, Janis, & Palmqvist, 2002; Perez‐Barberia & Gordon, 1999). A classic example of dietary constraint occurs among members of the genus *Equus*. Modern equids have continuously erupting, hypsodont (i.e., high crowned) cheek teeth (premolars and molars) that require constant wear, which is acquired through the introduction of exogenous grit into the mouth during grazing (Jardine, Janis, Sahney, & Benton, 2012). In the absence of sufficient wear, horses develop a deadly condition referred to as wave mouth, when the molars fail to wear at equal rates (Damuth & Janis, 2011; Janis & Fortelius, 1988). As a result, over their lifetimes, individuals are constrained into a grazing lifestyle (but note that they can exploit other resources for short periods of time). Due to their gritty diet, molars of adult *Equus* tend to have flat mesowear profiles and microwear dominated by abrasive features (i.e., scratches) (Fortelius & Solounias, 2000; Solounias & Semprebon, 2002).

The dentition of xenarthrans may be similarly constraining. The earliest xenarthrans were likely insectivores, and, like many other insectivorous mammals, evolved a simplified dentition or became completely edentulous (e.g., aardvarks, pangolins) (Gaudin & Croft, 2015; Vizcaíno, 2009). Within Pilosa, anteaters continue to persist on an insectivorous diet and are completely edentulous, whereas modern sloths have evolved into exclusively arboreal folivores (McNab, 1979). Having lost the ability to produce enamel, sloths instead rely on teeth composed of hypermineralized ever‐growing dentin (sometimes defined as “orthodentine”), which, like equids, also require consistent grinding against each other, food, and exogenous particulates to prevent overgrowth (Kalthoff, 2011; Ungar, Teaford, Glander, & Pastor, 1995). Although dentin is softer than enamel and thus exhibits wear in noncomparable ways (Hirschfeld, 1985; MacFadden, DeSantis, Labs Hochstein, & Kamenov, 2010), microwear studies have shown that xenarthran tooth wear is indicative of diet when compared within xenarthra (Haupt et al., 2013). We suggest that dietary constraints, such as are apparent among *Equus* species and sloths, are responsible for the high phylogenetic signal of mesowear and microwear.

There are a number of ways that tooth morphology influences and constrains the diets of individuals. Mammalian teeth vary widely in both form and function (Lucas, 2004; Reilly, McBrayer, & White, 2001; Wall & Smith, 2001). Teeth of different shapes are evolved to perform specific functions such as cutting, grinding, slicing, or pulverizing (Lucas, 2004). For example, grinding teeth are often characterized by numerous flat blades of enamel oriented perpendicular to the chewing stroke (Kaiser, Fickel, Streich, Hummel, & Clauss, 2010), while pulverizing teeth are shaped and function like a mortar and pestle (Lucas, 1979). Different tooth shapes have evolved to increase the effectiveness with which mammals orally process different food types. For example, carnivoran carnassial teeth perform slicing, allowing effective mastication of flesh, while lophodont equid molars perform grinding and thus effective processing of grasses (Lucas, 2004). While the evolution of specific tooth shapes improves mastication of particular foods, it can also limit the degree to which individuals can exploit other foods. For example, monocot leaves are characterized by numerous sclerenchymatous bundle sheaths and have thick cell walls (Wright & Illius, 1995). Significant grinding is required to exploit the nutrition in monocot cells. Carnivorans, particularly hypercarnivores (>70% meat in diet) such as felids (Van Valkenburgh, 2007), have reduced dentition and lack teeth with grinding surfaces (Solé & Ladevèze, 2017). Therefore, the shearing function of hypercarnivore carnassial teeth is insufficient for processing tough monocot leaves. Although less specialized carnivores can and do eat grasses, due, in part, to inefficient mastication, they are precluded from relying on monocots as their sole source of nutrition (considerations of gut morphology and excessive tooth wear notwithstanding). As such, through limiting the foods individual mammals can effectively orally process, tooth shape can limit the average diet of a species (Pineda‐Munoz et al., 2017). Furthermore, it is well established that tooth shape is phylogenetically conserved (Kangas, Evans, Thesleff, & Jernvall, 2004). We therefore expect members of the same species and of closely related species with similar tooth morphology to display similar dietary preferences (as shown in Table 1 and Figure 2a–b) and thus characteristics of their tooth wear.

Teeth of certain shapes may also influence wear through acting as guides during the chewing stroke (Kaiser et al., 2010; von Koenigswald, Anders, Engels, Schultz, & Kullmer, 2013). In mammal species with complex and high‐relief tooth cusps, the occlusal pathway is altered as the antagonistic teeth occlude (Maier, 1984). Tooth guidance is apparent as both attritional wear facets and striations or microwear oriented in the direction of tooth movement (von Koenigswald et al., 2013). Tooth guidance is less apparent among species with low‐relief cusps in which tooth occlusion is controlled primarily by the activity of the jaw‐closing muscles (Kaiser et al., 2010). The wear experienced by flatter teeth tends to be dominated by abrasive wear rather than attritional wear. Therefore, to some degree, guidance of the teeth by their antagonists or lack thereof during mastication influences the presence and distribution of certain types of wear. Given that tooth shape is phylogenetically conserved (Kangas et al., 2004), the degree of tooth guidance and, thus, the types of wear apparent on the teeth are expected to be similarly phylogenetically conserved.

Tooth wear is also influenced by the orientation of the chewing stroke, as it is determined by the organization of the chewing muscles (Fraser & Rybczynski, 2014; Lucas, 2004). The chewing stroke is divided into two phases, which correspond broadly to jaw closing and tooth incursion (phase I) and jaw opening and tooth excursion (phase II) (Greaves, 1980; Hiiemae, 2000; Janis, 1979; Rensberger, 1973; Wall & Smith, 2001). The angle of tooth incursion is influenced by the arrangement of the three primary jaw‐closing muscles, the masseter, pterygoideus, and temporalis. Grazing mammals, for example, emphasize transversely oriented shredding and shearing during chewing, in part because the origination surface for the superficial masseter is extended anterior to the zygomatic arch (Mendoza et al., 2002; Spencer, 1995). The result is the re‐orientation of the masseteric force arm from caudal to more rostral, allowing more muscular force to be applied at the molars in a buccolingual direction (DeMar & Barghusen, 1972; Greaves, 1991; Herring & Herring, 1974; Herring, Rafferty, Liu, & Marshall, 2001; Smith, 1993; Williams, Vinyard, Wall, & Hylander, 2007). Similarly, three‐dimensional reconstructions of carnivoran tooth occlusion show nonplanar attritional contact; the amount of lateral movement of the teeth during mastication is correlated with tooth number and dental complexity (Evans & Fortelius, 2008), both characters known to be highly phylogenetically conserved. As above, species with transversely oriented chewing strokes tend to show abrasive wear features. Thus, the orientation of the chewing stroke is also an influential factor in how the teeth wear, particularly in species with limited tooth guidance. Although there is likely some individual variation, it is unlikely that the orientation of the chewing muscles (areas of insertion and origination) is widely variable within a species or among closely related species that have inherited similar diets from their most recent common ancestor.

In summation, we suggest that both mesowear and microwear show high phylogenetic signal because the feeding apparatus of mammals is an evolutionary and functional module. Characteristics of the jaws, teeth, and chewing stroke evolve in concert to enable the exploitation of new food types (Fraser & Rybczynski, 2014). As a functional module, the different components of the feeding apparatus (e.g., jaws, teeth) must evolve and change together to maintain function or produce a change in function such as a transition from one feeding type to another (Raia, Carotenuto, Meloro, Piras, & Pushkina, 2010). For example, modern *Equus* have evolved high crowned cheek teeth as a means of increasing the lifespan of the teeth in the face of increased abrasive wear. But high crowned teeth require considerable space in the jaw. Modern *Equus* has therefore also evolved a robust and dorsoventrally wide mandible as well as posteriorly displaced orbits (Macfadden, 2000). A transition from brachydont to hypsodont could not have occurred in the absence of compensatory changes to the jaws. Furthermore, a shift to grazing on tough grasses that demand significant grinding required an associated shift in the orientation of the chewing stroke, the orientation of the enamel bands of the cheek teeth, and flattening of the tooth profile to enable a highly transverse chewing stroke (Fraser & Rybczynski, 2014). In turn, the ways in which *Equus* teeth experience wear are altered, trading attritional for abrasive wear and thus higher relief cusps for low relief.

In a similar vein, hypercarnivores such as felids require powerful jaw‐closing muscles for capturing and dispatching prey (Valkenburgh & Ruff, 1987). As a result, the temporalis muscle, a jaw‐closing muscle with a slightly posteriorly directed line of action, is comparatively large (Wall & Smith, 2001). The sagittal crest of the skull is similarly enlarged to provide a larger surface of origination for the temporalis. The result is a comparatively vertically oriented chewing stroke evolved for killing prey and cutting flesh (Valkenburgh & Ruff, 1987). The teeth of felids also show complex relief, enhanced tooth guidance during chewing, and prevalence of shearing‐type wear (Ungar, 2010). As in *Equus*, transitioning into a carnivorous niche has produced a suite of changes in the felid feeding apparatus. Slow changes in the function of the feeding apparatus and mammal diet on evolutionary timescales have thus combined to produce detectible phylogenetic signal in tooth wear proxies thought only to reflect much shorter timescales (i.e., the lifetime of individuals or last few meals).

### 4.1 Implications

Our finding of high phylogenetic signal for mesowear and microwear does not imply that the only information conveyed by tooth wear is phylogenetic; tooth wear is not necessarily taxonomy or phylogeny in disguise. The utility of mesowear and microwear for delineating individual and temporal differences in tooth wear is clear (Louys, Ditchfield, Meloro, Elton, & Bishop, 2012; Scott et al., 2005). Our interpretation is that the tooth wear of individuals from a particular species can vary within some range that is constrained by the evolutionary morphology of their feeding apparatus. As such, changes through time or among individuals can be detected but these changes will fall within the range set by morphology and thus the ways in which the feeding apparatus functions. In some cases, experimental diets produce changes in tooth wear but also induce wear features expected given the chewing mechanics of the species (Hoffman et al., 2015). Similarly, comparison of dentin wear among distantly related taxa with different dental morphologies suggests there is little basis for comparison (Haupt et al., 2013). We therefore do not intend to recommend the disuse of microwear and mesowear for understanding average species diets or more detailed aspects of their biology. But what do our findings mean in practice?

We do not suggest that the species, population, and individual‐level tooth wear differences reported in previous studies reflect phenomena other than chewing mechanics and diet. Our argument is a statistical one. Phylogenetic nonindependence can be a considerable problem if a comparison of distantly related taxa is desired (e.g., a comparison of *Equus* to *Bos*) and *p*‐values or exploratory methods such as discriminant function analysis are used to test for significant differences among trophic groups (Barr & Scott, 2014). We will not elaborate here because there is a vast literature on phylogenetic nonindependence. We highlight that compensations for phylogenetic nonindependence are rarely applied in tooth wear studies (Fraser et al., 2014; Mihlbachler & Solounias, 2006), despite the widespread use of phylogenetic comparative methods in ecology and paleontology (Blomberg & Garland, 2002; Losos, 2008; Miles & Dunham, 1993). We therefore recommend their application in studies of tooth wear wherever possible. For modern mammals, nearly complete phylogenies are widely available (Faurby & Svenning, 2015; Fritz & Purvis, 2010) using the taxonomy of both Wilson and Reeder (2005) and the International Union for Conservation of Nature, respectively. For fossil mammals, the number of phylogenies is increasing, cladistic and otherwise (Fraser et al., 2015), and in the absence of phylogenetic information, taxonomy can also be used (Soul & Friedman, 2015). There are numerous phylogenetic comparative methods that can be put to good use by scientists using tooth wear including, but not limited to, phylogenetically independent contrasts, phylogenetic generalized least squares, phylogenetic principal components analysis, and PDFA. All of these analyses are now available in the free software platform R in packages such as ape, geiger, and caper (Harmon et al., 2008; Orme et al., 2011; Paradis, 2006).

## 5 SUMMARY

- Mammal diets and tooth wear show significant phylogenetic signal when using Pagel's λ and phylogenetic heritability (*H* ^2^).
- We suggest that phylogenetic conservation of functional traits related to feeding explains phylogenetic signal in mammal diet and tooth wear.
- The ways in which teeth wear are determined, in part, by dietary constraints placed on animals by their morphology and the influence of morphology on the ways in which the teeth occlude.
- Tooth wear is not merely phylogeny in disguise; it can vary among individuals and time periods, but the degree of variation is constrained by morphology and thus phylogeny.
- Application of common, well‐documented phylogenetic comparative methods can circumvent statistical issues related to nonindependence among tooth wear data points due to PNC.
- Restriction of tooth wear comparisons to closely related species with similar dental morphologies will reduce the need for phylogenetic comparative methods but limit within‐guild comparison of distantly related taxa.

## CONFLICT OF INTEREST

None declared.

## AUTHOR CONTRIBUTIONS

Danielle Fraser conceived and designed the study, compiled published data from the literature, carried out the statistical analyses, interpreted the statistical analyses, and wrote the manuscript. Ryan J. Haupt compiled published data from the literature and wrote the manuscript. W. Andrew Barr helped conceive and design the study, helped design figures, interpreted the statistical results, and wrote the manuscript.
