# Supplementary figures and images for: Phylogenetic signal in tooth wear dietary niche proxies
Source: Ecol Evol. 2018 May 3;8(11):5355–68. doi: 10.1002/ece3.4052 (PMC6010706; doi:10.1002/ece3.4052)

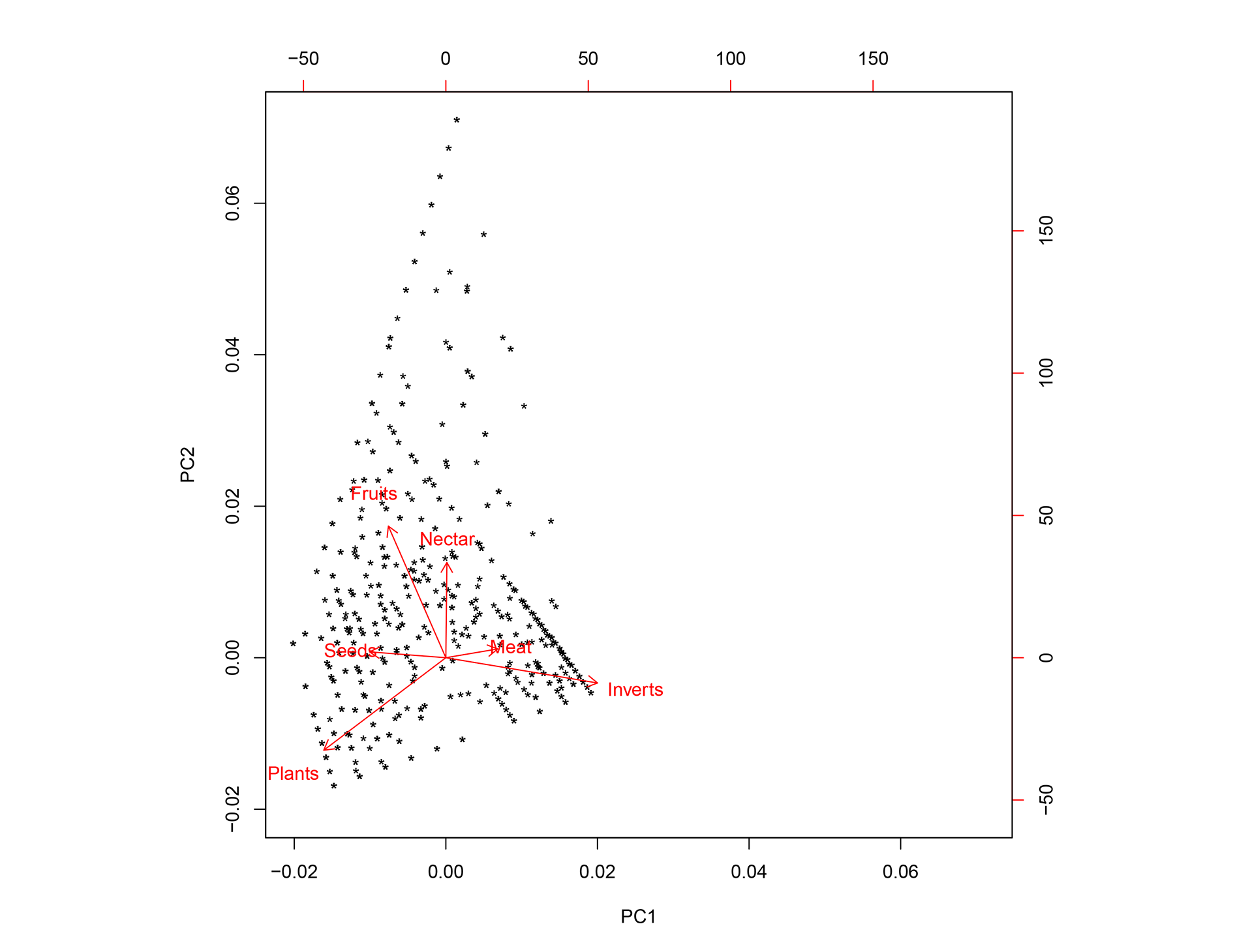

Supplement: Supplementary file 1 [file ECE3-8-5355-s001.tif]

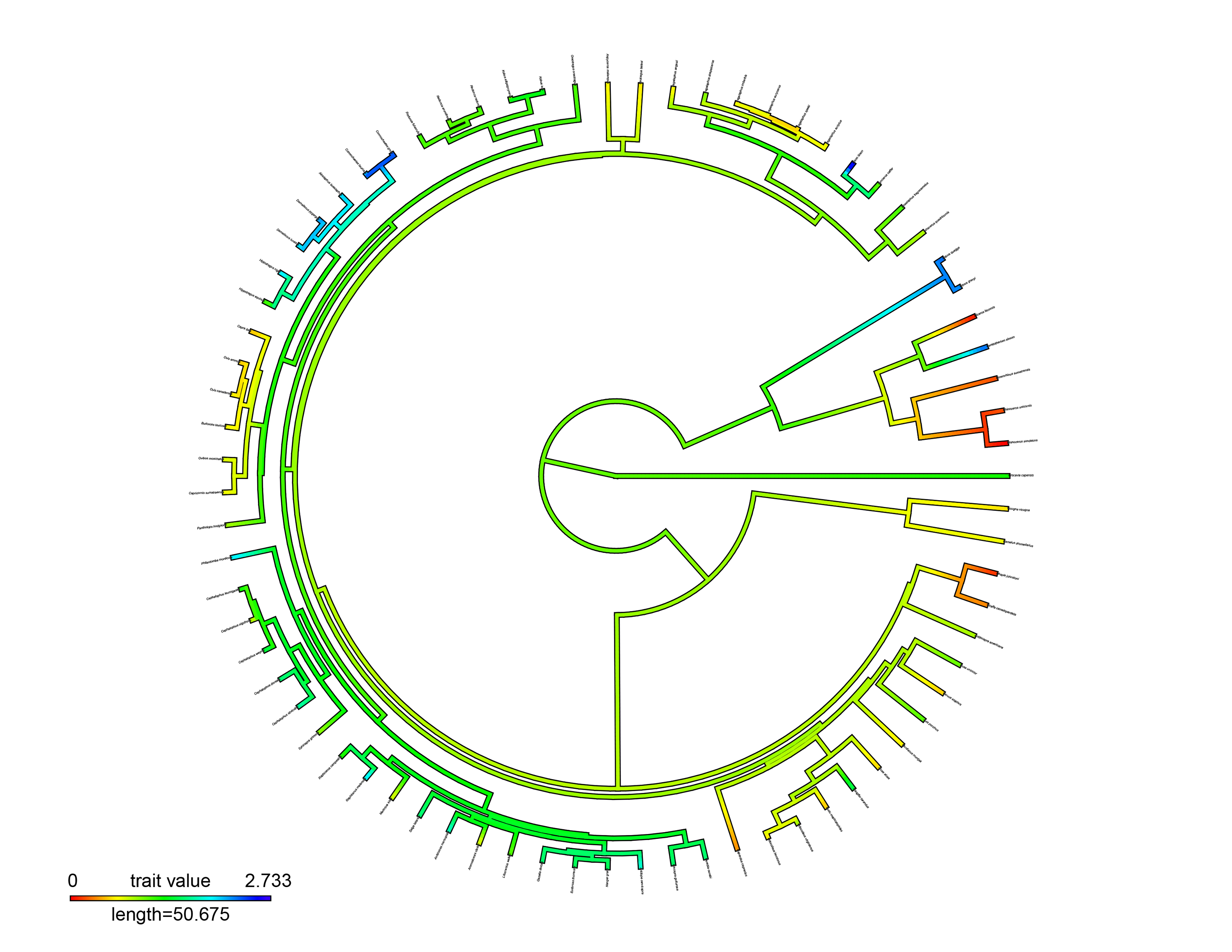

Supplement: Supplementary file 2 [file ECE3-8-5355-s002.tif]

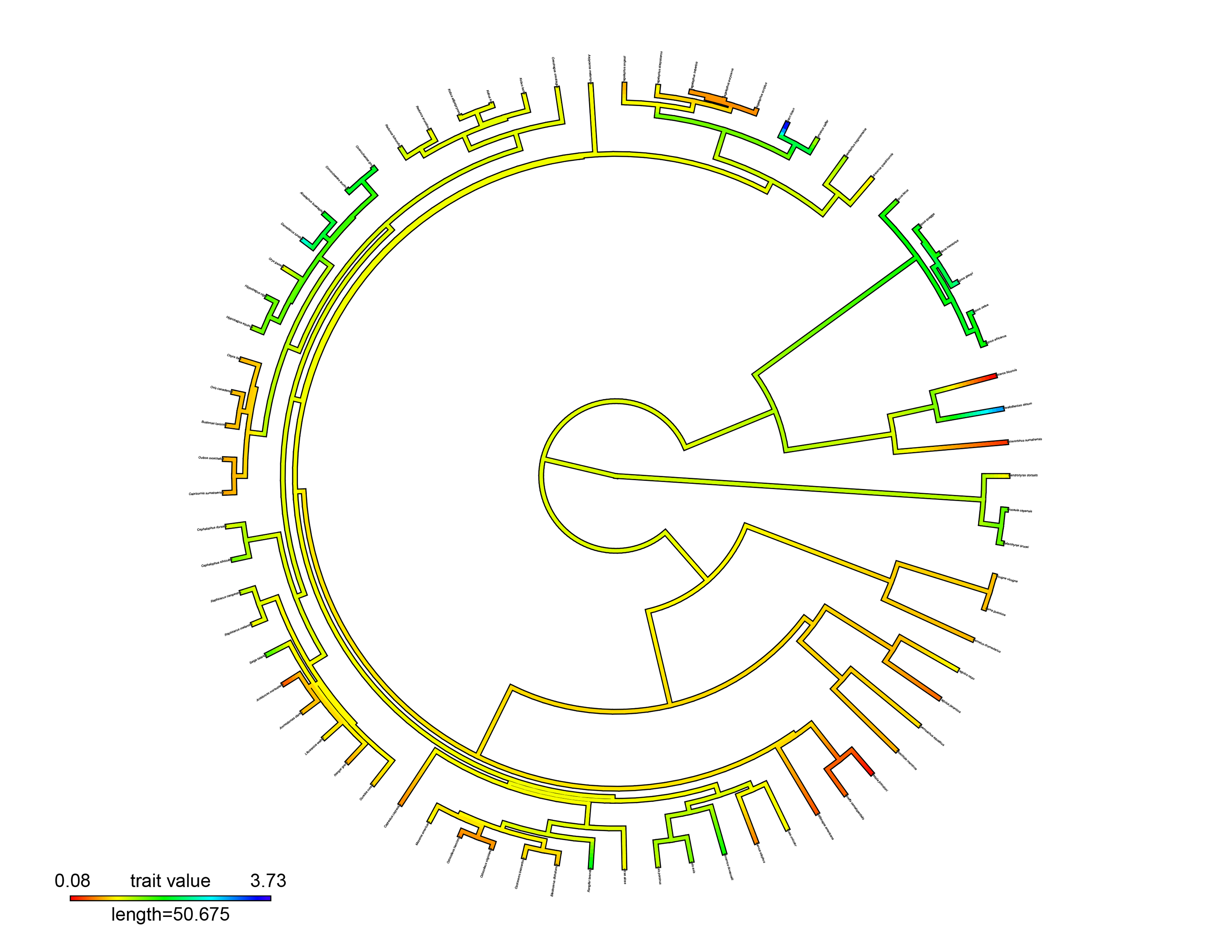

Supplement: Supplementary file 3 [file ECE3-8-5355-s003.tif]

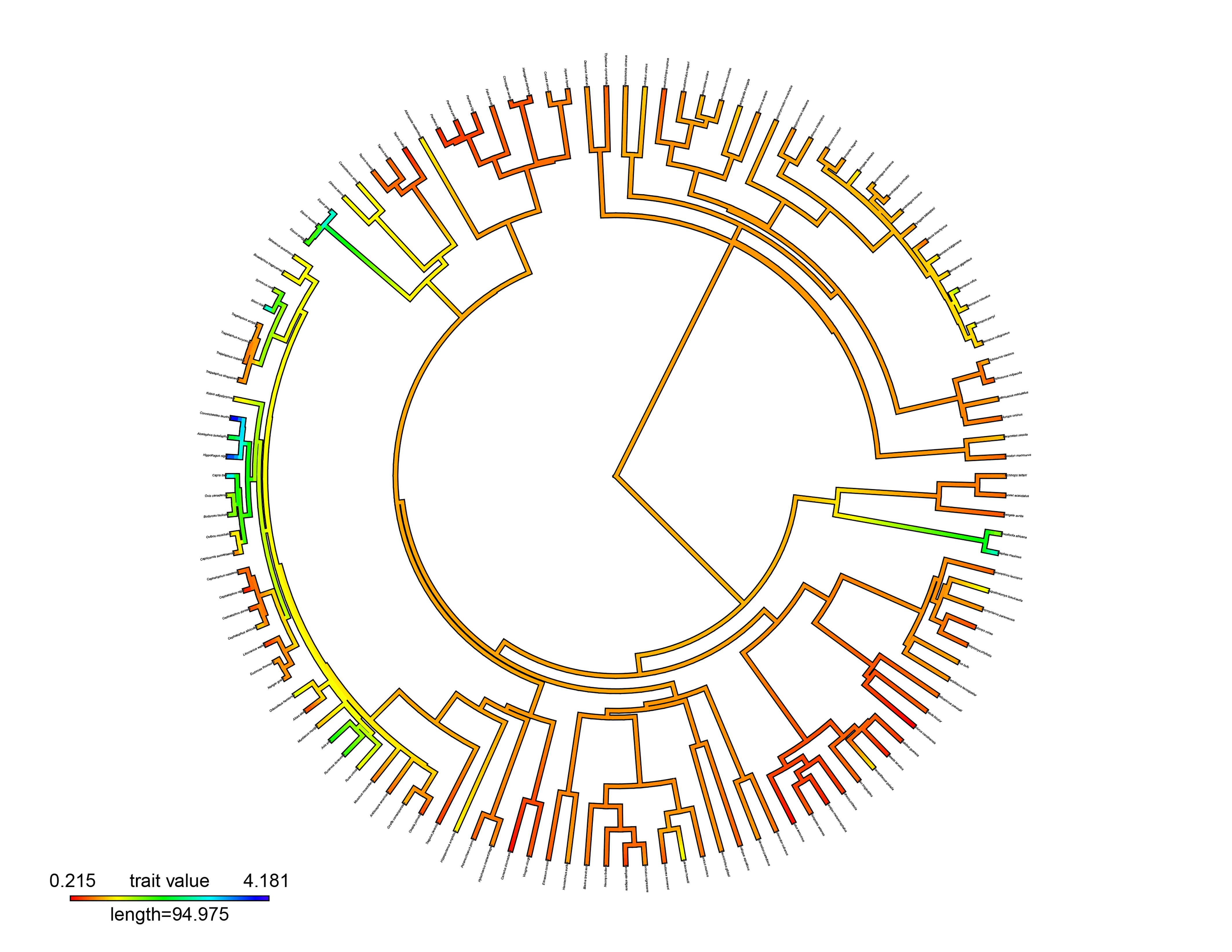

Supplement: Supplementary file 4 [file ECE3-8-5355-s004.tif]

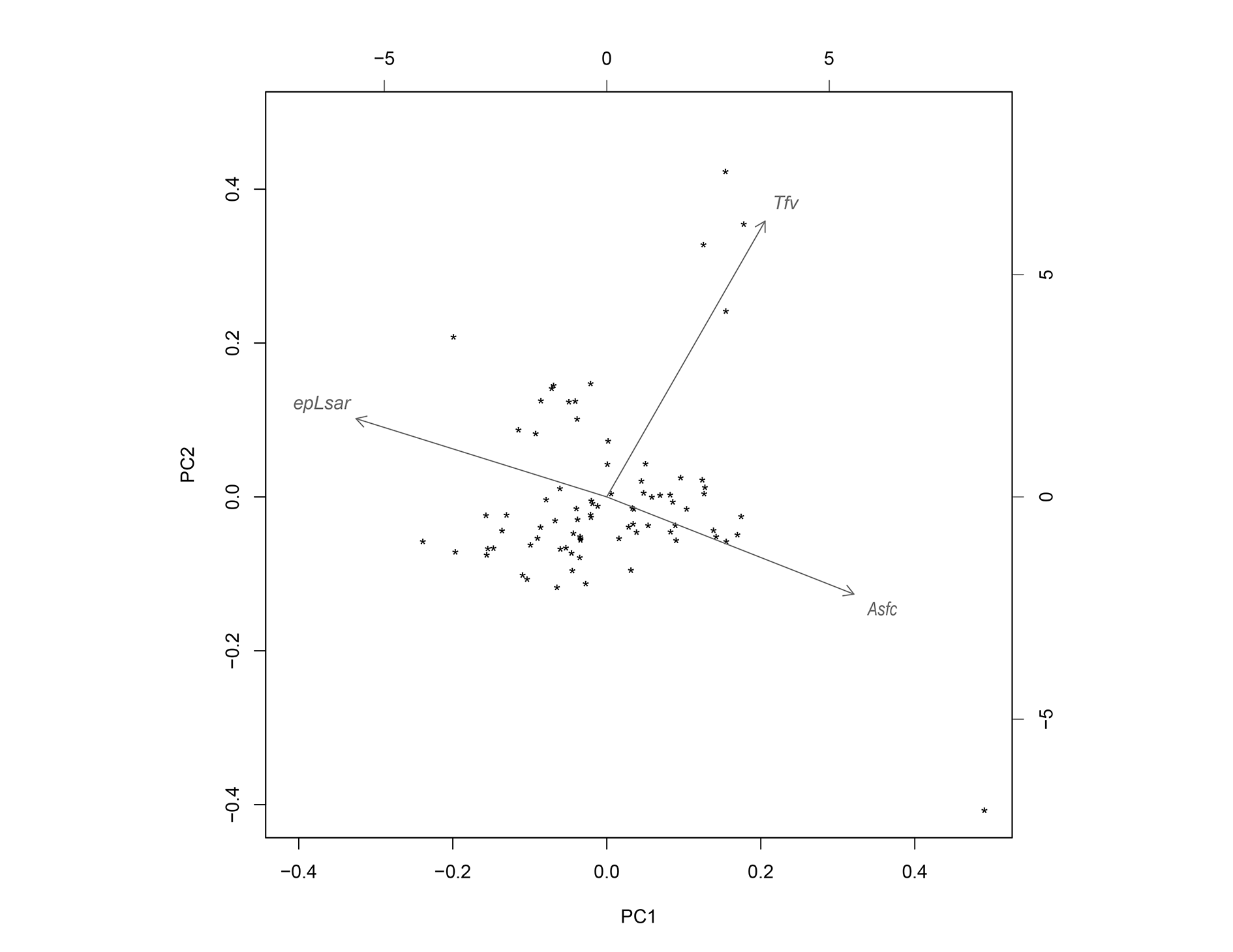

Supplement: Supplementary file 5 [file ECE3-8-5355-s005.tif]

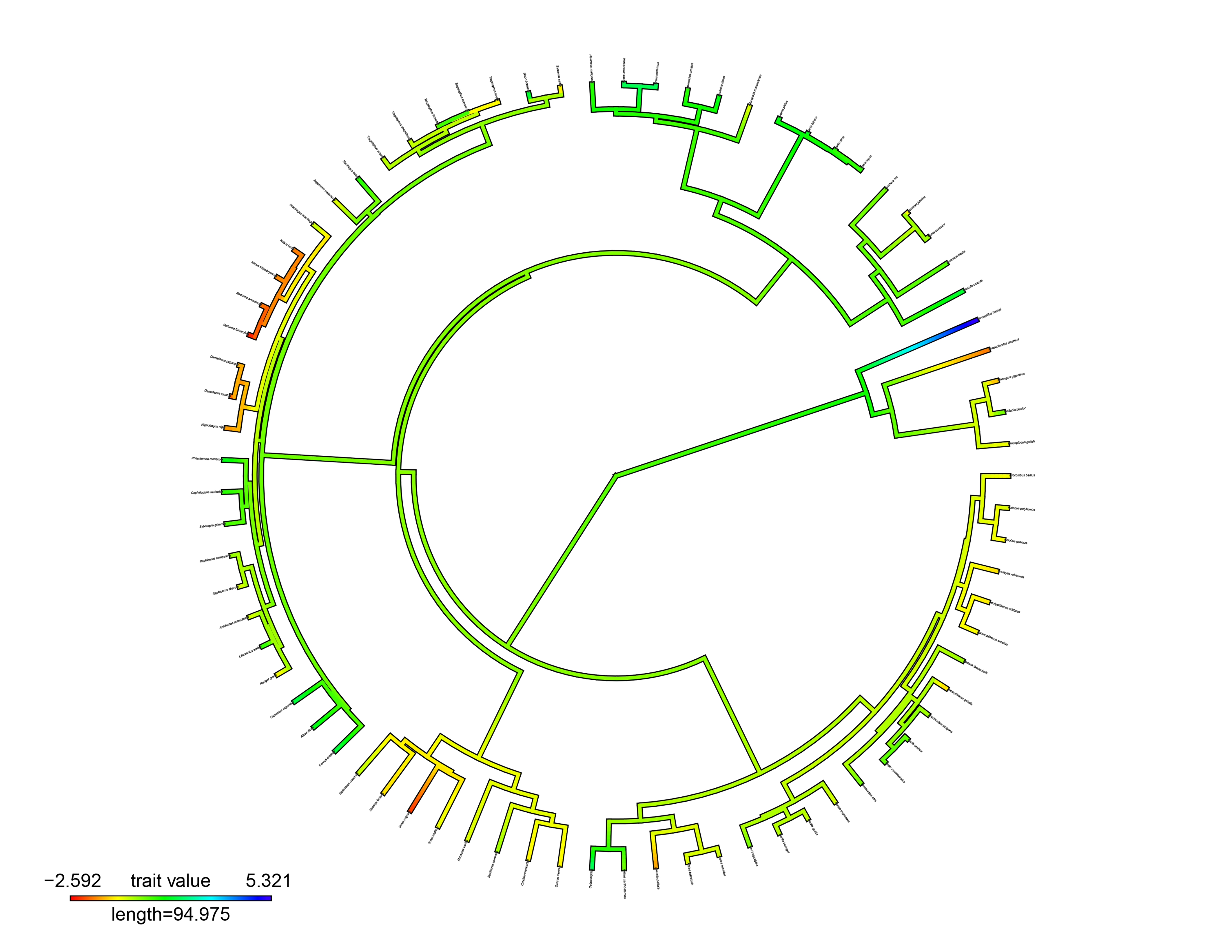

Supplement: Supplementary file 6 [file ECE3-8-5355-s006.tif]

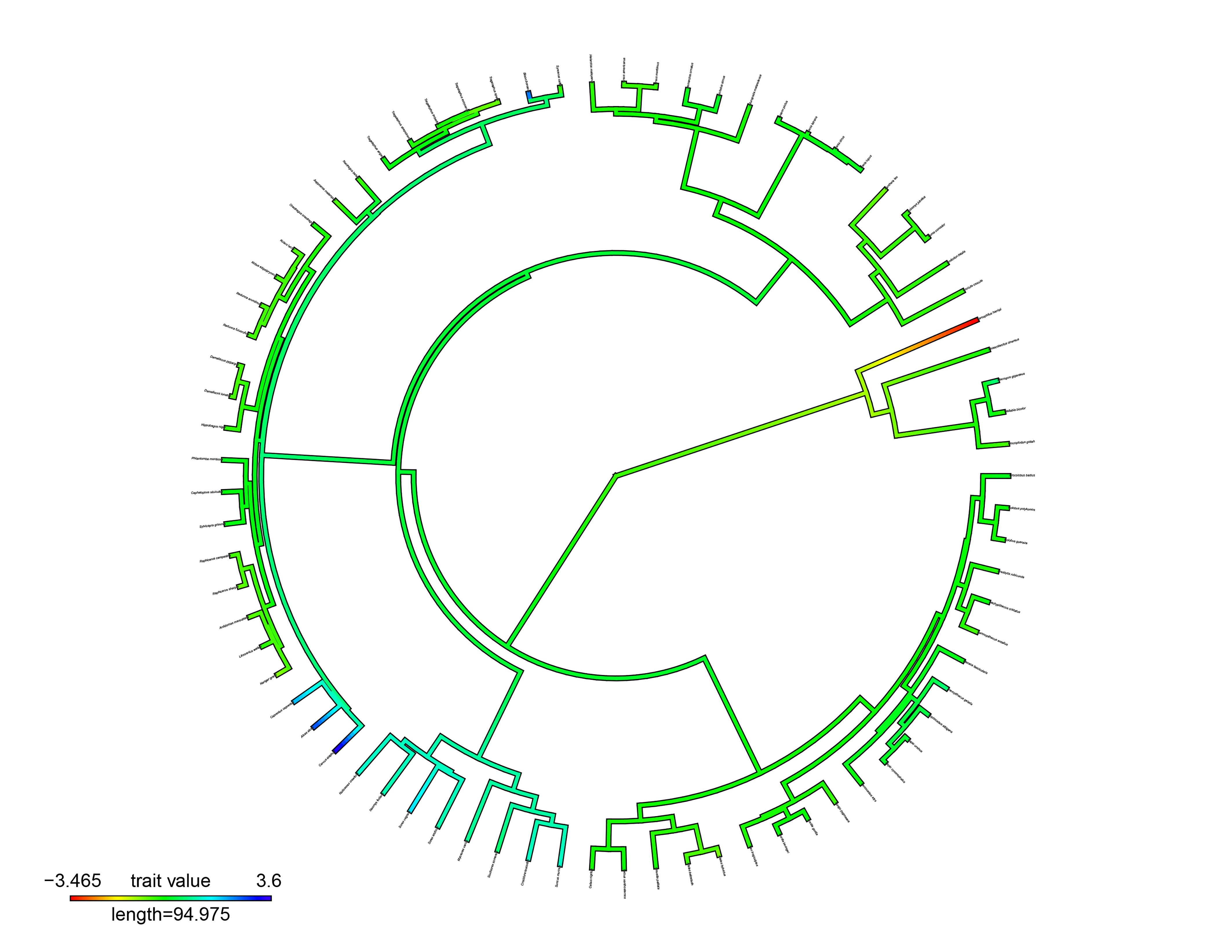

Supplement: Supplementary file 7 [file ECE3-8-5355-s007.tif]
